# Comparative Analysis of Polycyclic Aromatic Hydrocarbons and Halogenated Polycyclic Aromatic Hydrocarbons in Different Parts of *Perilla frutescens* (L.) Britt.

**DOI:** 10.3390/molecules27103133

**Published:** 2022-05-13

**Authors:** Pengfei Wang, Bo Jin, Chaojie Lian, Kaijing Guo, Chen Ma

**Affiliations:** 1 Institute of Materia Medica, Chinese Academy of Medical Sciences & Peking Union Medical College, Beijing 100050, China; pengfeiwang@imm.ac.cn (P.W.); jinboyws@imm.ac.cn (B.J.); guokaijing@imm.ac.cn (K.G.); 2 National Institutes for Food and Drug Control, Beijing 102627, China; lianchaojie@nifdc.org.cn

**Keywords:** *Perilla frutescens* (L.) Britt., PAHs, HPAHs, GC-MS, ILCR

## Abstract

*Perilla frutescens* (L.) Britt., a medicinal herb and edible plant, is very popular among East Asian countries. The perilla leaves, stems and seeds can be used as traditional medicines and foods. Polycyclic aromatic hydrocarbons (PAHs) and halogenated PAHs (HPAHs) are organic pollutants that are widely present in the environment, such as in water, air and soil, and are harmful to humans. In this study, the contents of 16 PAHs and 4 HPAHs in perilla leaves, stems and seeds were determined by gas chromatography tandem mass spectrometry (GC-MS). A total of 12 PAHs were detected in all samples, and no HPAHs were detected. The total contents of PAHs in perilla leaves, stems and seeds varied from 41.93 to 415.60 ng/g, 7.02 to 51.52 ng/g and 15.24 to 180.00 ng/g, respectively. The statistical analyses showed that there were significant differences in the distribution of PAHs in perilla leaves, stems and seeds. On the basis of the toxic equivalent quantity (TEQ) and incremental lifetime cancer risk (ILCR) model, the cancer risks of the intake of perilla leaves, stems and seeds were assessed to be from 3.30 × 10^−8^ to 2.11 × 10^−5^, 5.52 × 10^−9^ to 5.50 × 10^−8^ and 1.20 × 10^−8^ to 1.41 × 10^−7^, respectively. These were lower than 10^−4^ (the priority risk level of the EPA) and suggested that there may be almost no cancer risk from the intake of these traditional Chinese medicines (TCMs).

## 1. Introduction

*Perilla frutescens* (L.) Britt. (*P. frutescens*), belonging to the *Lamiaceae* family, is a kind of annual herb. Because of its high edible and medicinal value, it is widely cultivated in Asian countries such as China, Japan, Vietnam, Korea and Laos [1,2,3]. Fresh perilla leaves are often eaten as vegetables, spices or food colorants. Perilla seeds are an important source of perilla seed oil, which has the highest content of ω-3 fatty acids [4]. In addition to edible usages, perilla leaves, stems and seeds are also traditional Chinese medicines (TCMs) with different therapeutic effects that are recommended by the Chinese Pharmacopoeia and can be combined with different TCMs to form prescriptions for various therapeutics [5]. Perilla plants contain a variety of chemical compounds, such as flavonoids, volatile oils, triterpenes, fatty acids, phenolic compounds, etc. Pharmacological studies have shown that perilla leaves and seeds possess antidepressant, antioxidant, antitumor and hypolipidemic activities [6,7,8,9]. Perilla stems have the effect of relieving pain and preventing miscarriage [10]. However, for TCMs, attention not only needs to be given to their bioactive constituents and pharmaceutical values but also to the residual toxic and harmful pollutants that might be absorbed by the human body.

Polycyclic aromatic hydrocarbons (PAHs) are ubiquitous organic contaminants. The International Agency for Research on Cancer (IARC) classified some PAHs as probable or possibly carcinogens to humans [11]. Sixteen PAHs have been identified as priority pollutants by the United States Environmental Protection Agency (US EPA). Halogenated polycyclic aromatic hydrocarbons (HPAHs), byproducts of human activities, are products in which hydrogen atoms in PAHs are replaced by chlorine atoms or bromine atoms [12]. Similarly to their parent PAHs, HPAHs are also carcinogenic because of their capacities to combine and activate the aryl hydrocarbon receptor (AhR) and induce DNA damage [13,14]. A great number of studies have reported that PAHs and HPAHs are widespread in the environment, such as in water, air, soil, and sediments [15,16,17]. Additionally, PAHs and HPAHs have been found in many kinds of food, including honey, milk, milk powders and seafood products [18,19,20].

For a long time, the problem of pollutant residues has been the focus of quality control of TCMs. There have been some studies indicating that PAHs have been found in TCMs. Ishizaki et al. [21] determined 15 kinds of PAHs in five tea products and 29 herbal products by using the automated online in-tube solid-phase microextraction/HPLC-FLD method. Yan et al. [22] enriched seven kinds of PAHs from four kinds of leafy traditional Chinese medicines by using cholesterol-functionalized magnetic nanoparticles. Yu et al. [23] successfully determined 16 EPA PAHs in 32 TCMs of different types, including roots, flowers, stems, fruits, seeds, and leaves. However, to date, information on PAHs and HPAHs in different medicinal parts of *P. frutescens* has not been available. Taking the above factor into consideration, the leaves, stems and seeds of *P. frutescens* were targeted as the research objects, and the pollution levels of sixteen EPA PAHs and four HPAHs were investigated. Moreover, the differences in contamination status between different medicinal parts were evaluated, and the potential sources of contaminants were explored. Ultimately, we assessed the potential health risks from the intake of PAHs by consuming perilla leaves, stems and seeds.

## 2. Results and Discussion

### 2.1. Method Optimization and Validation

The Optimization of GC-MS Conditions. The fragment information of target compounds was obtained in full scan mode, and 3–4 characteristic ions were selected for SIM mode. The mass spectrometry parameters are shown in Table 1 for qualitative and quantitative analysis. In order to ensure that the target compounds were separated and not affected by interference components, different temperature programs were optimized. Finally, the GC-MS method was determined to detect the target compounds.

Preparation methods for samples were optimized. Different medicinal parts contain different interference components and thus have different matrix categories. Pigments are the main interference component in the extraction of leaves and stems. The extraction efficiencies of target compounds by *n*-hexane, acetonitrile and ethyl acetate were similar, and the pigments of leaves and stems in *n*-hexane extracts were less. Therefore, *n*-hexane was used as the extraction solvent for leaves and stems. The volume of *n*-hexane (10, 20 and 30 mL), sonication time (10, 20 and 30 min) and type of adsorbent for purification (PSA, GCB and C18) were optimized for extraction of target compounds in leaves and stems. Perilla seeds contain a large amount of perilla oil, and *n*-hexane has high solubility for oil. Acetonitrile was used as the extraction solvent, and the interference of oil was reduced. The SPE columns were better than the adsorbent in the purification of oil. The volume of acetonitrile (5, 10 and 20 mL) and the type of SPE column (PSA, C18 and Florisil) were optimized. Acetonitrile was selected as the extraction solvent for seeds, and Florisil and C18 SPE columns were used for purification.

Method validation: Each calibration level was spiked with 20 ng/mL of internal standard. The calibration curves for PAHs and HPAHs were linear over the range 1-fold LOQ–100-fold LOQ of standard solutions (r > 0.99). At low (4 LOQ), medium (10 LOQ) and high (50 LOQ) concentration levels, the recoveries for 16 PAHs and 4 HPAHs in perilla leaves, stems and seeds ranged from 51.55% to 154.10%, and the RSD ranged from 0.58% to 28.10%. The validation results for the method are summarized in Tables S1–S3 (Supplementary Materials).

### 2.2. Comparative Analysis

**Figure 1:**
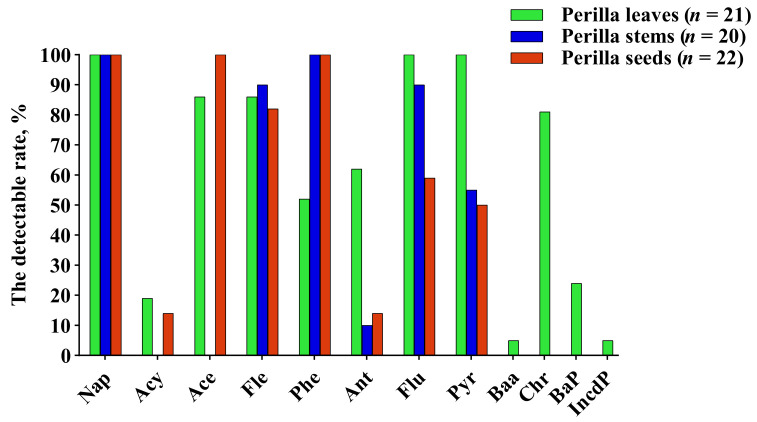
The detectable rate of a single PAH in perilla leaves, stems and seeds.

The contents of PAHs and HPAHs in perilla leaves, stems and seeds were determined by an internal standard calibration curve method using deuterated isotope compounds as internal standard. Table S4 (Supplementary Materials) lists the contents of individual and total PAHs in perilla leaves, stems and seeds. A total of 12 kinds of PAHs were detected in all samples of *P. frutescens*, and no HPAHs were detected. Figure 1 shows the detectable rate of individual PAHs in the samples. Among the 12 kinds of PAHs detected, Nap, Ace, Acy, Fle, Ant, Phe, BaA, Flu, Pyr and Chr were lower molecular weight PAHs (LMW PAHs) with two to four rings, and BaP and IncdP were higher molecular weight PAHs (HMW PAHs) with five to six rings. The abovementioned PAHs were also all detected in perilla leaves. Eight and six kinds of LMW PAHs were detected in perilla seed and stem samples, respectively. Compared with HMW PAHs, LMW PAHs were detected more frequently. It has been suggested that the uptake depends on the physicochemical properties of PAHs, and those LMW PAHs that are more volatile are more preferentially absorbed [24]. For the single PAH congener detected, the detectable rate in the three medicinal parts of *P. frutescens* varied. Nap was detected in all samples. Fle was detected in more than 80% of samples of perilla leaves, stems and seeds. Ace’s detection rate was more than 85% in perilla leaves and perilla seeds but not in perilla stems. The detectable rates of Phe, Flu and Pyr were 52%, 100% and 100% in perilla leaves, respectively, and 100%, more than 50% and 50 % in perilla stems and seeds, respectively. In perilla leaves, BaP was detected in 24% of samples, and BaA and IncdP were detected in very few samples.

**Figure 2:**
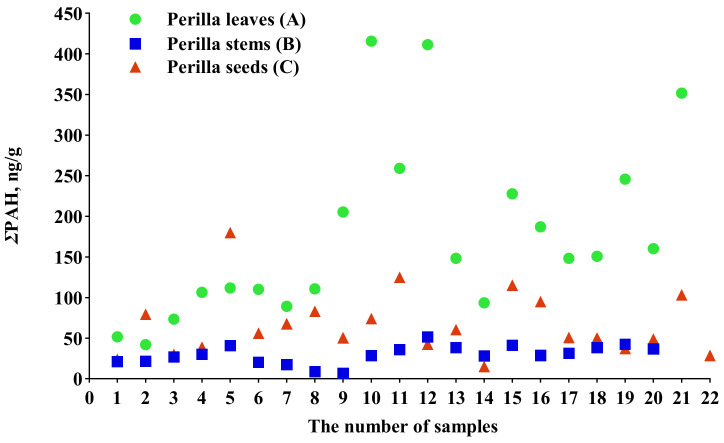
ΣPAHs in each sample.

The total contents of PAHs (ΣPAHs) in perilla leaves, stems and seeds varied from 41.93 to 415.60 ng/g, 7.02 to 51.52 ng/g and 15.24 to 180.00 ng/g, respectively. The ΣPAHs in each sample are shown in Figure 2. The status of PAH contamination varied among samples of the same medicinal part of *P. frutescens.* This might be related to the growth environment. Some studies have indicated that two ways of transferring PAHs from the environment to plants exist [24,25]. PAHs in the air could be accumulated in plants by the stomata of the leaves, which might be the principal pathway [24]. Second, PAHs accumulated from the soil to plants through roots could also be passively transported to leaves driven by transpiration flux [25,26]. The differences in PAH pollution levels in environmental media of the same area and the characteristics of different medicinal parts of plants cause significant differences in ΣPAHs in different medicinal parts of *P. frutescens* (*p* < 0.05). In this study, the mean ΣPAHs in perilla leaves (176.20 ± 109.00 ng/g) were higher than those in perilla stems (29.75 ± 11.44 ng/g) and perilla seeds (66.14 ± 39.00 ng/g). This may be related to the fact that the surface area of leaves exposed to the air is larger than that of stems and seeds. Additionally, as shown in this study, the contamination of PAHs in seeds seemed more serious than in stems. These results might be related to the composition of *P. frutescens* and the properties of PAHs. Perilla seeds with a high lipid content might contribute to the accumulation of lipophilic PAHs [26,27].

**Figure 3:**
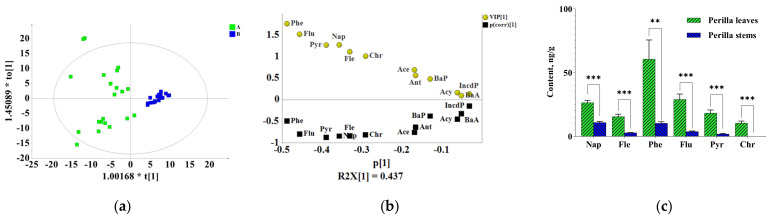
The difference between perilla leaves (A) and stems (B). (**a**) OPLS-DA score plot; (**b**) (V + S)-plot of OPLS-DA. (**c**) The contents of differential compounds (*C*, ng/g) (**: *p* ≤ 0.01; ***: *p* ≤ 0.001).

**Figure 4:**
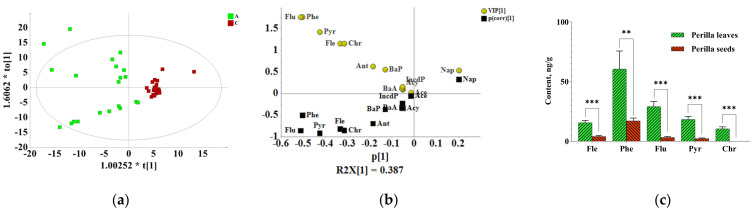
The difference between perilla leaves (A) and seeds (C). (**a**) OPLS-DA score plot; (**b**) (V + S)-plot of OPLS-DA. (**c**) The contents of differential compounds (*C*, ng/g) (**: *p* ≤ 0.01; ***: *p* ≤ 0.001).

**Figure 5:**
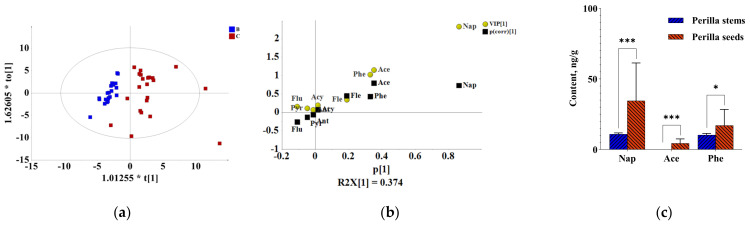
The difference between perilla stems (B) and seeds (C). (**a**) OPLS-DA score plot; (**b**) (V + S)-plot of OPLS-DA. (**c**) The contents of differential compounds (*C*, ng/g) (*: *p* ≤ 0.05; ***: *p* ≤ 0.001).

To investigate which detected components exhibited the major contribution to the difference in different medicinal parts of *P. frutescens*, multivariate principal component analysis (PCA) and orthogonal partial least-squares discrimination analysis (OPLS-DA) were carried out. PCA is an unsupervised model recognition technique to describe the structure of the dataset and provides results about similarities and differences between samples without knowing anything about them [28]. PCA was performed on the PAH content data from all samples. The PCA score plot showed a separation among the perilla leave, stem and seed sample groups, which indicated the differences in contamination status among them. To explore the underlying differences, OPLS-DA, which is a supervised multivariate data analysis, was used for analysis. In this study, OPLS-DA was performed to enhance the sample separation observed in PCAs and identify the compounds that provided the most relevant variables for differences among the three groups of samples [29]. The OPLS-DA, which emphasized the differences between two groups, showed that there was a clear separation between the different medicinal parts, which indicated a significant difference in the contamination status between the different medicinal parts (Figure 3a, Figure 4a and Figure 5a). Differential pollutants were screened according to the OPLS-DA score plot combined with the VIP value and the *t* test *p* value (VIP value > 1, *p* value < 0.05) [30]. The significant difference between perilla leaves and stems was reflected in Nap, Fle, Pyr, Phe, Chr and Flu (Figure 3b). The significant difference between perilla leaves and seeds was reflected in Fle, Pyr, Phe, Flu and Chr (Figure 4b). The difference between perilla stems and seeds was reflected in Nap, Phe and Ace (Figure 5b). The average contents of all these compounds contributing to significant differences are shown in Figure 3c, Figure 4c and Figure 5c. These compounds followed the same trend as the ΣPAHs, that is, *C* _(perilla leaves)_ > *C* _(perilla seeds)_ > *C* _(perilla stems)_.

On the whole, Figure 2Figure 3a, Figure 4a and Figure 5a also showed the intragroup contamination status of PAHs. The samples of perilla seeds and stems clustered together well, which indicated that the intragroup difference in the PAH contamination status of the two parts was small, while the distribution of perilla leaves was relatively dispersed, which showed that the intragroup difference was large. This may be related to the morphological characteristics of leaves.

### 2.3. Health Risk Assessment

BaP is considered the most toxic and carcinogenic PAH and is classified as a carcinogen to humans (Group 1) by the IARC [11]. The toxic equivalency factor (TEF) of PAHs in carcinogenicity is determined by referring to the carcinogenic efficacy of the PAH homologue BaP, and the carcinogenic effect of BaP is considered to be 1 [31]. Since the toxic equivalent quantity (TEQ) can be regarded as a better indicator of effective toxicity than content, we calculated the TEQ of PAHs. For the toxicity of the samples, the TEQ was calculated as the sum of the TEQ_i_ values of the individual PAHs (Equation (1)) [32]. The TEQ_i_ value was calculated for each PAH content in the sample and the corresponding TEF (TEF_PAHi_) from the literature [31], so that:

TEQ = ΣTEQ_i_ = Σ(C_PAHi_ × TEF_PAHi_) (1)

As seen in Table 2, the TEQ values of perilla leaf samples numbered 11, 16–19 and 21 were relatively large, mainly because of the detection of IncdP or BaP. This condition also indicated that once HMW PAHs are detected in the sample, their toxicity equivalents are generally the major contributors to the TEQ.

To evaluate the relationship between carcinogenic risk and the consumption of perilla leaves, stems and seeds, the following formula (Equation (2)) can be used to calculate the incremental lifetime cancer risk (ILCR) [33]:

ILCR = TEQ × IR × CF × ED × CSF/(BW × AT)(2)

The CSF value for gastrointestinal intake is 7.3 (mg/kg-day)^−1^ [34]. IR is the intake of these herbs per day (g/day). CF is the unit conversion factor, 10^−6^ kg/mg. ED is the exposure period, and the conventional exposure period from adulthood is 50 years. BW is body weight, generally taken as 60 kg. AT is the average length of life for carcinogens, 77.4 in China (based on World Health Statistics 2021, WHO [35]).

In addition, the leaves, seeds and stems of *P. frutescens* are three medicinal materials in the Chinese Pharmacopoeia that are regulated by the administration and dosage (up to a limit of 10 g per day). We can assess the excess cancer risk caused by exposure to PAHs in the diet through the consumption of these three medicinal materials. The ILCRs associated with dietary exposure to ΣPAHs for Chinese adults ranged from 3.30 × 10^−8^ to 2.11 × 10^−5^, 5.52 × 10^−9^ to 5.50 × 10^−8^ and 1.20 × 10^−8^ to 1.41 × 10^−7^, respectively, for the perilla leaves, seeds and stems. According to permissible limits or acceptable risk levels defined by the US EPA, the acceptable risk level is 10^−6^, and the priority risk level is 10^−4^ [20,36]. The present results showed that the ILCRs through intake of perilla stems and seeds were lower than the acceptable risk level. Additionally, the ILCRs of perilla leaves that included HMW PAHs may be lower than the priority risk level but higher than the acceptable risk level. These results indicate that more attention needs to be given to the pollution status of PAHs in perilla leaves. Furthermore, when perilla leaves, stems and seeds are used for medicinal purposes, the transfer rate of PAHs from medicinal materials to medicinal infusions needs to be considered. Zachara et al. [37] found that the transfer rate of Σ4PAHs (BaP, BaA, BbF and Chr) from tea to tea infusion was 0.48~1.72%. The edible method of TCMs is similar to that of tea. It can be inferred that the amount of PAHs in perilla leaves, stems or seeds entering the immersion liquid was lower, and therefore, the ILCRs of the intake of these TCMs might be lower.

## 3. Materials and Methods

### 3.1. Materials and Reagents

Acetonitrile and *n*-hexane were HPLC grade and purchased from Honeywell International, Inc. (Charlotte, NC, USA). *N*-(*n*-Propyl) ethylenediamine (PSA) sorbent, Florisil SPE column (500 mg, 6 mL) and C18 SPE column (500 mg, 6 mL) were purchased from ANPEL Laboratory Technologies, Inc. (Shanghai, China). The Florisil SPE column and C18 SPE column were primed with 6 mL of acetonitrile prior to use. Analytical-grade anhydrous magnesium sulfate was purchased from Fuchen Chemical Reagent Co., Ltd. (Tianjin, China).

The 16 EPA PAHs and four HPAHs (Table 1) were purchased from Dr. Ehrenstorfer GmbH (Augsburg, Germany) or MedChemExpress LLC (Monmouth Junction, NJ, USA). The purities of these compounds are above 97%.

The commercial solution of Nap-d_8_, Phe-d_10_, Ace-d_10_, Chr-d_12_ and Per-d_12_ (2.00 mg/mL, dichloromethane) was obtained from o2si smart solutions (Charleston, SC, USA). It was diluted to 200 ng/mL with *n*-hexane as an internal standard solution.

A total of 63 samples, consisting of 21 perilla leaves, 20 perilla stems and 22 perilla seeds, were collected from different origins in China (Table S4, Supplementary Materials). In this study, samples of perilla leaf were numbered A1 to A21, samples of perilla stem were numbered B1 to B20, and samples of perilla seed were numbered C1 to C22.

All glassware was carefully rinsed with solvents before use and finally baked in a hot air oven at 150 °C.

### 3.2. GC-MS Conditions

The contents of PAHs and HPAHs in perilla leaves, stems and seeds were determined by GC-MS with a 7890A gas chromatograph interfaced with a 5975C mass spectrometer (Agilent Technologies, Inc., Santa Clara, CA, USA) using an HP-5MS capillary column (30 m × 0.25 mm × 0.25 μm). One microliter of the sample was injected in pulsed splitless mode. The injector temperature was 290 °C. The column oven temperature was programmed as follows: 60 °C maintained for 2.0 min, increased to 160 °C at 5 °C/min, ramped to 210 °C at 10 °C/min, then to 255 °C at 2 °C/min, increased to 300 °C at 4 °C/min, and held for 10.0 min. Helium gas (99.99%) was used as the carrier gas, and the flow rate was 1.0 mL/min. The temperature of the transfer line was 290 °C. The temperature of the ion source was 230 °C, and the quadrupole temperature was 150 °C. The solvent delay time was 10.0 min. The mass spectrometer was operated in EI mode (70 eV). Quantification was performed under selected ion-monitoring (SIM) mode. The SIM parameters used for the 20 target compounds and internal standards are shown in detail in Table 1.

### 3.3. Sample Preparation

#### 3.3.1. The Preparation of Perilla Leaves and Stems

The samples were powdered and screened through 355 μm sieves. Each sample of powder (1.00 g) was accurately weighed and placed into a glass centrifuge tube. Then, 200 μL of internal standard solution (200 ng/mL) was added to the tube. Then, 20 mL of *n*-hexane was added. The tube was vortexed intensively for 30 s, sonicated for 10 min, and then centrifuged for 5 min (3000 rpm). Afterwards, 15 mL of the upper layer was transferred into a glass centrifuge tube that included a sorbent of 150 mg PSA and 500 mg MgSO_4_. The extract was again vortexed vigorously for 30 s, sonicated for 10 min, and then centrifuged for 5 min (3000 rpm). Finally, 10 mL of the upper layer was concentrated to near dryness by rotary evaporation at 35 °C. The residue was dissolved in 1.0 mL of *n*-hexane before GC-MS analysis.

#### 3.3.2. The Preparation of Perilla Seeds

The samples were powdered. Each sample (1.00 g) was accurately weighed and placed into a glass centrifuge tube. Then, 200 μL of internal standard solution (200 ng/mL) was added to the tube. Then, 10 mL of acetonitrile was added. The tube was vortexed intensively for 30 s, sonicated for 10 min, and then centrifuged for 5 min (3000 rpm). Afterwards, 5 mL of the upper layer was first purified on a Florisil column using 6 mL and then 1 mL acetonitrile. The eluent was further cleaned up on a C18 column. Finally, the target compound in the C18 column was eluted with 6 mL of acetonitrile. The collected eluent was evaporated to near dryness. The residue was dissolved in 1.0 mL of *n*-hexane before GC-MS analysis.

### 3.4. Method Verification

Method validation was carried out with limit of quantification (LOQ), linearity, accuracy and precision. Analytical standards were dissolved in *n*-hexane to prepare stock standard solutions of individual standards (C_PAHi_ ≈ 40 μg/mL, C_HPAHi_ ≈ 400 μg/mL). The LOQ was calculated as 10 times the signal-to-noise ratio. The calibration curves for PAHs and HPAHs were established, and each calibration level contained 20 ng/mL of internal standard. The mixed standard solutions of PAHs and HPAHs and internal standard were spiked into a sample matrix of perilla leaves, stems and seeds to determine the recoveries (R%) and the relative standard deviation (RSD%).

### 3.5. Statistical Analysis

Normality tests, Kruskal–Wallis tests and *t* tests were carried out using SPSS Statistics 26.0 (IBM, Armonk, NY, USA) to assess the statistical significance of differences. The differences were considered significant at a probability of 0.05 or lower (*p* ≤ 0.05). SIMCA (MKS Data Analytics Solutions, San Jose, CA, USA) provided the information on contributors to the differences in contamination of the three medicinal parts of *P. frutescens*.

## 4. Conclusions

In this study, the contamination status of 16 kinds of PAHs and 4 kinds of HPAHs in different medicinal parts of *P. frutescens* was analyzed. In all 63 samples, 12 kinds of PAHs were detected, and no HPAHs were detected. Except for BaP and IncdP detected in a small minority of perilla leaves, the detected PAHs were lower molecular weight (2–4 rings). The contamination of PAHs in perilla leaves, stems and seeds was significantly different. The highest levels of ΣPAHs were found in leaves (176.20 ± 109.00 ng/g), followed by seeds (66.14 ± 39.00 ng/g) and stems (29.75 ± 11.44 ng/g). That was related not only to the content of PAHs in the environment but also to the characteristics of different parts of the plant. The ILCR values showed that the consumption of these different medicinal parts of *P. frutescens* may not cause any health problems. It is worth noting that HMW PAHs such as BaP and IncdP have a greater contribution of TEQ to ILCR and need more attention. Moreover, considering that perilla leaves, seeds or stems need to be taken in prescriptions with other TCMs and that other TCMs may also be polluted by PAHs to different degrees, the specific risks of the intake of prescriptions need further research.

## Figures and Tables

**Table 1:** The retention time (Rt), quantitative ion and qualitative ion of each compound.

Name	Abbreviation	Rt (min)	Quantitative Ion	Qualitative Ion	Quantitative Internal Standard
Naphthalene-d_8_	Nap-d_8_	12.4	136.1	137.1, 108.0	\
Naphthalene	Nap	12.5	128.0	127.0, 101.9	Nap-d_8_
Phenanthrene-d_10_	Ace-d_10_	20.3	164.1	162.1, 160.1	\
Acenaphthylene	Acy	19.6	152.0	151.0, 76.0, 63.0	Ace-d_10_
Acenaphthene	Ace	20.5	154.0	152.0, 76.0	Ace-d_10_
Fluorene	Fle	22.8	166.0	165.0, 139.0	Ace-d_10_
Acenaphthene-d_10_	Phe-d_10_	26.0	188.1	160.1, 94.1	\
Phenanthrene	Phe	26.1	178.1	89.1, 152.0	Phe-d_10_
Anthracene	Ant	26.3	178.1	89.1, 152.0	Phe-d_10_
2-Bromofluorene	2-BrFle	27.8	165.0	244.0, 245.9, 162.9	Phe-d_10_
2,7-Dichlorofluorene	2,7-Cl_2_Fle	29.5	199.0	234.0, 162.9, 200.9	Phe-d_10_
Fluoranthene	Flu	30.1	202.0	200.0, 100.9, 88.0	Phe-d_10_
Pyrene	Pyr	31.0	202.0	100.9, 200.0	Phe-d_10_
9-Bromoanthracene	9-BrAnt	31.1	258.0	177.0, 176.0, 88.0	Phe-d_10_
Chrysene-d_12_	Chr-d_12_	37.9	240.1	236.1, 120.1	\
9,10-Dibromoanthracene	9,10-Br_2_Ant	37.7	336.0	176.0, 88.1	Chr-d_12_
Benzo(a)anthracene	Baa	37.9	228.0	225.9, 114.1	Chr-d_12_
Chrysene	Chr	38.1	228.0	225.9, 114.1	Chr-d_12_
Perylene-d_12_	Per-d_12_	48.9	264.1	260.1, 265.1	\
Benzo(b)fluoranthene	BbF	46.0	252.0	250.1, 126.0, 112.8	Per-d_12_
Benzo(k)fluoranthene	BkF	46.2	252.0	250.1, 126.0, 112.8	Per-d_12_
Benzo(a)pyrene	BaP	48.4	252.0	250.0, 126.0	Per-d_12_
Indeno(1,2,3-c,d)pyrene	IncdP	56.0	276.0	138.0, 277.0	Per-d_12_
Dibenzo(a,h)anthracene	DahA	56.3	278.0	279.0, 138.8	Per-d_12_
Benzo(g,h,i)perylene	BghiP	57.2	276.0	137.1, 138.0, 274.0	Per-d_12_

**Table 2:** The results of TEQ (ng/g) and ILCR for all samples.

NO.	Perilla Leaves (A)		Perilla Stems (B)		Perilla Seeds (C)	
	TEQ	ILCR	TEQ	ILCR	TEQ	ILCR
1	0.05	4.05 × 10^−8^	0.02	1.66 × 10^−8^	0.02	1.88 × 10^−8^
2	0.04	3.30 × 10^−8^	0.02	1.68 × 10^−8^	0.08	6.24 × 10^−8^
3	0.17	1.32 × 10^−7^	0.03	2.13 × 10^−8^	0.03	2.34 × 10^−8^
4	0.18	1.45 × 10^−7^	0.03	2.36 × 10^−8^	0.04	3.06 × 10^−8^
5	0.22	1.70 × 10^−7^	0.04	3.21 × 10^−8^	0.18	1.41 × 10^−7^
6	0.19	1.53 × 10^−7^	0.02	1.60 × 10^−8^	0.06	4.42 × 10^−8^
7	0.09	7.01 × 10^−8^	0.02	1.36 × 10^−8^	0.07	5.31 × 10^−8^
8	0.30	2.38 × 10^−7^	0.01	6.82 × 10^−9^	0.08	6.51 × 10^−8^
9	0.38	2.97 × 10^−7^	0.01	5.52 × 10^−9^	0.05	3.97 × 10^−8^
10	0.67	5.29 × 10^−7^	0.03	2.23 × 10^−8^	0.07	5.81 × 10^−8^
11	2.28	1.79 × 10^−6^	0.04	2.80 × 10^−8^	0.12	9.81 × 10^−8^
12	0.64	5.02 × 10^−7^	0.07	5.50 × 10^−8^	0.04	3.35 × 10^−8^
13	0.25	2.00 × 10^−7^	0.04	3.02 × 10^−8^	0.06	4.75 × 10^−8^
14	0.09	7.35 × 10^−8^	0.03	2.21 × 10^−8^	0.02	1.20 × 10^−8^
15	0.36	2.84 × 10^−7^	0.04	3.24 × 10^−8^	0.14	1.11 × 10^−7^
16	18.18	1.43 × 10^−5^	0.03	2.25 × 10^−8^	0.12	9.15 × 10^−8^
17	19.22	1.51 × 10^−5^	0.03	2.47 × 10^−8^	0.05	3.98 × 10^−8^
18	19.15	1.50 × 10^−5^	0.06	4.46 × 10^−8^	0.05	3.94 × 10^−8^
19	20.37	1.60 × 10^−5^	0.04	3.32 × 10^−8^	0.04	2.93 × 10^−8^
20	0.29	2.29 × 10^−7^	0.04	2.90 × 10^−8^	0.05	3.83 × 10^−8^
21	26.89	2.11 × 10^−5^	-	-	0.12	9.65 × 10^−8^
22	-	-	-	-	0.03	2.25 × 10^−8^

## Data Availability

The data presented in this study are available on request from the corresponding author.

## Supplementary Materials

The following supporting information can be downloaded at: https://www.mdpi.com/article/10.3390/molecules27103133/s1, Table S1: Method verification experimental parameters for 20 target compounds in perilla leaves; Table S2: Method verification experimental parameters for 20 target compounds in perilla stems; Table S3: Method verification experimental parameters for 20 target compounds in perilla seeds; Table S4: Contents of polycyclic aromatic hydrocarbons in perilla leaves, stems and seeds (ng/g).

## Sample Availability

Samples of the compounds are not available from the authors.
