# Supplementary material for: Comparative Analysis of Polycyclic Aromatic Hydrocarbons and Halogenated Polycyclic Aromatic Hydrocarbons in Different Parts of Perilla frutescens (L.) Britt
Source: Molecules. 2022 May 13;27(10):3133. doi: 10.3390/molecules27103133 (PMC9145503; doi:10.3390/molecules27103133)
Supplement: Supplementary file 1 [file molecules-27-03133-s001.zip › molecules-1699346-supplementary.pdf]

*molecules*

MDPI

*Supplementary Materials*

# Comparative Analysis of Polycyclic Aromatic Hydrocarbons and Halogenated Polycyclic Aromatic Hydrocarbons in Different Parts of *Perilla frutescens* (L.) Britt.

**Pengfei Wang [1,†], Bo Jin [1,†], Chaojie Lian [2], Kaijing Guo [1] and Chen Ma [1,*]**

1 Institute of Materia Medica, Chinese Academy of Medical Sciences & Peking Union Medical College, Beijing 100050, China; pengfeiwang@imm.ac.cn (P.W.); jinboyws@imm.ac.cn (B.J.); guokaijing@imm.ac.cn (K.G.)
2 National Institutes for Food and Drug Control, Beijing 102627, China; lianchaojie@nifdc.org.cn
* Correspondence: mach@imm.ac.cn; Tel.: +86-010-63165239
† These authors are equally contributed to this work.

**Table S1.** Method verification experimental parameters for 20 target compounds in perilla leaves.

| Name | LOQ (ng/mL) | range (ng/mL) | Curve | r | low | | medium | | high | |
|---|---|---|---|---|---|---|---|---|---|---|
| | | | | | R*% | RSD* % | R*% | RSD* % | R*% | RSD* % |
| Nap | 1.00 | 1.00~100.0 | Y=1.198x+0.01233 | 1.0000 | 94.63 | 13.84 | 91.17 | 7.06 | 97.47 | 2.23 |
| Acy | 1.00 | 1.00~100.0 | Y=1.795x-0.01077 | 0.9995 | 98.43 | 3.60 | 82.34 | 5.73 | 71.45 | 5.13 |
| Ace | 1.06 | 1.06~106.0 | Y=1.057x+0.0003974 | 0.9995 | 100.52 | 4.22 | 92.92 | 2.25 | 86.64 | 2.17 |
| Fle | 1.02 | 1.02~102.0 | Y=1.277x-0.001937 | 0.9995 | 92.97 | 5.38 | 87.74 | 4.59 | 88.25 | 3.28 |
| Phe | 1.05 | 1.05~105.0 | Y=1.043x+0.01077 | 1.0000 | 101.00 | 28.10 | 101.70 | 11.63 | 94.40 | 3.62 |
| Ant | 1.06 | 1.06~106.0 | Y=1.082x-0.002735 | 1.0000 | 99.39 | 5.33 | 70.74 | 13.61 | 51.55 | 18.45 |
| Flu | 1.04 | 1.04~104.0 | Y=0.8615x+0.002241 | 1.0000 | 119.60 | 6.69 | 99.14 | 6.99 | 96.76 | 1.72 |
| Pyr | 1.03 | 1.03~103.0 | Y=0.9357x+0.003122 | 1.0000 | 109.76 | 5.39 | 94.11 | 4.16 | 94.13 | 1.33 |
| Baa | 4.20 | 4.20~420.0 | Y=1.074x-0.02391 | 0.9995 | 96.65 | 1.40 | 85.07 | 1.64 | 81.68 | 1.50 |
| Chr | 4.08 | 4.08~408.0 | Y=1.097x-0.03729 | 0.9995 | 91.49 | 2.63 | 85.05 | 1.52 | 86.49 | 2.27 |
| BbF | 8.16 | 8.16~816.0 | Y=1.487x-0.2526 | 0.9985 | 110.47 | 4.54 | 113.40 | 7.29 | 124.35 | 5.59 |
| BkF | 8.48 | 8.48~848.0 | Y=1.213x-0.2562 | 0.9980 | 118.16 | 3.58 | 113.19 | 7.07 | 118.69 | 4.90 |
| BaP | 8.32 | 8.32~832.0 | Y=1.253x-0.2124 | 0.9985 | 114.75 | 2.01 | 101.70 | 2.52 | 94.34 | 1.27 |
| IncdP | 8.40 | 8.40~840.0 | Y=1.319x-0.2194 | 0.9980 | 103.56 | 4.85 | 103.19 | 6.43 | 110.42 | 5.09 |
| DahA | 8.40 | 8.40~840.0 | Y=1.331x-0.2529 | 0.9980 | 110.02 | 5.26 | 110.48 | 7.04 | 116.69 | 5.09 |
| BghiP | 8.08 | 8.08~808.0 | Y=1.398x-0.1827 | 0.9990 | 97.65 | 4.28 | 96.85 | 6.43 | 106.32 | 6.09 |
| 2-BrFle | 2.01 | 2.01~201.0 | Y=0.4691x+0.0009647 | 0.9995 | 112.86 | 9.02 | 94.19 | 7.48 | 90.42 | 9.13 |
| 2,7-$Cl_2$Fle | 1.996 | 1.996~199.6 | Y=0.4318x+0.0004611 | 1.0000 | 113.30 | 2.35 | 101.09 | 2.63 | 96.69 | 1.67 |
| 9-BrAnt | 2.01 | 2.01~201.0 | Y=0.2952x-+0.0004537 | 0.9995 | 102.82 | 5.49 | 86.64 | 6.79 | 73.15 | 7.82 |
| 9,10-$Br_2$Ant | 4.032 | 4.032~403.2 | Y=0.2911x-0.0002582 | 0.9995 | 100.71 | 0.79 | 90.63 | 1.91 | 89.00 | 1.69 |

* R is recovery and RSD is relative standard deviation (n=6).

**Table S2.** Method verification experimental parameters for 20 target compounds in perilla stems.

| Name | LOQ (ng/mL) | range (ng/mL) | Curve | r | low | | medium | | high | |
|---|---|---|---|---|---|---|---|---|---|---|
| | | | | | R*% | RSD*% | R*% | RSD*% | R*% | RSD*% |
| Nap | 1.00 | 1.00~100.0 | Y=1.27x+0.01382 | 1.0000 | 98.57 | 3.70 | 97.64 | 6.69 | 94.48 | 2.26 |
| Acy | 1.00 | 1.00~100.0 | Y=1.602x-0.01554 | 1.0000 | 101.21 | 4.63 | 99.09 | 2.76 | 95.84 | 3.88 |
| Ace | 1.06 | 1.06~106.0 | Y=1.096x-0.004665 | 1.0000 | 99.39 | 2.17 | 99.02 | 1.43 | 95.91 | 2.08 |
| Fle | 1.02 | 1.02~102.0 | Y=1.296x-0.005373 | 0.9995 | 110.59 | 4.73 | 110.92 | 3.96 | 109.86 | 3.35 |
| Phe | 1.05 | 1.05~105.0 | Y=1.073x+0.002883 | 0.9995 | 100.39 | 13.03 | 95.28 | 3.23 | 97.09 | 1.57 |
| Ant | 1.06 | 1.06~106.0 | Y=0.9527x-0.003097 | 0.9995 | 113.01 | 4.29 | 112.23 | 3.44 | 111.35 | 2.96 |
| Flu | 1.04 | 1.04~104.0 | Y=0.7866x+0.002176 | 0.9995 | 118.54 | 3.31 | 123.11 | 2.94 | 118.72 | 2.96 |
| Pyr | 1.03 | 1.03~103.0 | Y=0.8520x+0.00165 | 0.9995 | 122.15 | 2.58 | 123.48 | 3.02 | 121.72 | 3.42 |
| Baa | 4.20 | 4.20~420.0 | Y=0.9021x-0.03493 | 0.9990 | 111.98 | 2.38 | 115.89 | 1.88 | 109.51 | 1.58 |
| Chr | 4.08 | 4.08~408.0 | Y=1.088x-0.02432 | 0.9995 | 103.46 | 1.69 | 101.37 | 1.78 | 95.38 | 1.54 |
| BbF | 8.16 | 8.16~816.0 | Y=2.331x-0.6011 | 0.9965 | 83.64 | 1.51 | 79.50 | 1.80 | 72.12 | 1.79 |
| BkF | 8.48 | 8.48~848.0 | Y=1.714x-0.6188 | 0.9940 | 86.56 | 1.51 | 79.12 | 3.05 | 69.09 | 1.99 |
| BaP | 8.32 | 8.32~832.0 | Y=1.555x-0.5014 | 0.9935 | 104.88 | 2.04 | 105.09 | 1.80 | 85.16 | 1.82 |
| IncdP | 8.40 | 8.40~840.0 | Y=1.535x-0.5101 | 0.9930 | 100.95 | 1.84 | 102.75 | 0.58 | 92.51 | 1.86 |
| DahA | 8.40 | 8.40~840.0 | Y=1.589x-0.5900 | 0.9925 | 94.30 | 2.67 | 94.20 | 1.01 | 83.29 | 3.52 |
| BghiP | 8.08 | 8.08~808.0 | Y=1.984x-0.5968 | 0.9945 | 91.68 | 1.98 | 89.97 | 0.80 | 77.51 | 2.34 |
| 2-BrFle | 2.01 | 2.01~201.0 | Y=0.3931x+0.001018 | 0.9990 | 124.95 | 2.02 | 126.53 | 3.23 | 125.63 | 1.86 |
| 2,7-$Cl_2$Fle | 1.996 | 1.996~199.6 | Y=0.3265x+0.0003083 | 0.9995 | 137.42 | 2.68 | 142.03 | 2.91 | 142.34 | 2.56 |
| 9-BrAnt | 2.01 | 2.01~201.0 | Y=0.2239x-0.001743 | 0.9990 | 134.86 | 1.88 | 139.96 | 3.53 | 139.74 | 2.93 |
| 9,10-$Br_2$Ant | 4.032 | 4.032~403.2 | Y=0.2300x+0.01002 | 1.0000 | 110.58 | 2.28 | 114.49 | 2.48 | 118.86 | 1.83 |

* R is recovery and RSD is relative standard deviation (n=6).

**Table S3.** Method verification experimental parameters for 20 target compounds in perilla seeds.

| Name | LOQ(ng/mL) | range (ng/mL) | Curve | r | low | | medium | | high | |
|---|---|---|---|---|---|---|---|---|---|---|
| | | | | | R*% | RSD* % | R*% | RSD* % | R*% | RSD* % |
| Nap | 1.00 | 1.00~100.0 | Y=1.383x+0.001455 | 1.0000 | 100.38 | 15.75 | 154.10 | 6.53 | 101.81 | 4.23 |
| Acy | 1.00 | 1.00~100.0 | Y=2.229x-0.01153 | 0.9995 | 91.55 | 7.99 | 76.32 | 6.33 | 73.89 | 5.35 |
| Ace | 1.06 | 1.06~106.0 | Y=1.297x-0.01778 | 1.0000 | 84.52 | 7.88 | 69.73 | 6.89 | 66.28 | 5.35 |
| Fle | 1.02 | 1.02~102.0 | Y=1.519x-0.02516 | 0.9990 | 104.76 | 14.79 | 79.78 | 12.04 | 75.01 | 5.85 |
| Phe | 1.05 | 1.05~105.0 | Y=1.134x-0.01012 | 0.9990 | 119.29 | 12.40 | 135.64 | 7.54 | 107.93 | 3.36 |
| Ant | 1.06 | 1.06~106.0 | Y=1.177x-0.04884 | 0.9970 | 123.13 | 8.43 | 120.91 | 14.91 | 125.00 | 4.47 |
| Flu | 1.04 | 1.04~104.0 | Y=1.146x-0.0004075 | 1.0000 | 117.06 | 6.86 | 115.94 | 3.08 | 118.91 | 3.19 |
| Pyr | 1.03 | 1.03~103.0 | Y=1.226x-0.002019 | 0.9995 | 110.77 | 5.64 | 106.93 | 2.71 | 119.55 | 3.13 |
| Baa | 4.20 | 4.20~420.0 | Y=1.091x-0.05862 | 0.9985 | 110.74 | 0.95 | 127.53 | 1.33 | 116.96 | 2.28 |
| Chr | 4.08 | 4.08~408.0 | Y=1.201x-0.116 | 0.9990 | 89.90 | 4.51 | 91.92 | 4.90 | 92.57 | 2.20 |
| BbF | 8.16 | 8.16~816.0 | Y=2.892x-1.086 | 0.9950 | 70.70 | 7.13 | 85.83 | 7.75 | 54.77 | 3.07 |
| BkF | 8.48 | 8.48~848.0 | Y=1.77x-0.6211 | 0.9915 | 102.33 | 4.97 | 103.65 | 9.75 | 55.43 | 3.11 |
| BaP | 8.32 | 8.32~832.0 | Y=1.819x-0.6602 | 0.9930 | 93.88 | 6.04 | 89.90 | 1.60 | 64.87 | 2.96 |
| IncdP | 8.40 | 8.40~840.0 | Y=1.951x-0.7619 | 0.9930 | 96.86 | 5.26 | 108.73 | 9.88 | 73.68 | 3.25 |
| DahA | 8.40 | 8.40~840.0 | Y=1.814x-0.7238 | 0.9925 | 107.95 | 5.20 | 118.00 | 9.20 | 87.29 | 3.22 |
| BghiP | 8.08 | 8.08~808.0 | Y=2.132x-0.4871 | 0.9975 | 70.01 | 5.77 | 75.77 | 8.55 | 61.69 | 3.26 |
| 2-BrFle | 2.01 | 2.01~201.0 | Y=0.5304x-0.004628 | 0.9990 | 129.97 | 2.35 | 97.29 | 1.30 | 124.74 | 2.49 |
| 2,7-$Cl_2$Fle | 1.996 | 1.996~199.6 | Y=0.4702x-0.009168 | 0.9995 | 147.04 | 2.81 | 137.79 | 1.46 | 146.78 | 2.90 |
| 9-BrAnt | 2.01 | 2.01~201.0 | Y=0.3181x-0.002983 | 0.9995 | 113.76 | 8.79 | 103.42 | 7.83 | 125.10 | 4.44 |
| 9,10-$Br_2$Ant | 4.032 | 4.032~403.2 | Y=0.3287x+0.002506 | 0.9995 | 60.12 | 3.63 | 69.99 | 1.95 | 82.43 | 3.27 |

* R is recovery and RSD is relative standard deviation (n=6).

**Table S4.** Contents of polycyclic aromatic hydrocarbons in perilla leaves, stems and seeds (ng/g).

| Parts | No | Origin | Nap | Acy | Ace | Fle | Phe | Ant | Flu | Pyr | BaA | Chr | BaP | IncdP | ΣPAHs |
|---|---|---|---|---|---|---|---|---|---|---|---|---|---|---|---|
| Perilla leaves | A1 | Anhui | 24.74 | - | 5.76 | - | - | - | 13.11 | 7.89 | - | - | - | - | 51.50 |
| | A2 | Heilongjiang | 25.08 | - | - | - | - | - | 10.81 | 6.04 | - | - | - | - | 41.93 |
| | A3 | Sichuan | 33.04 | - | 4.20 | - | - | - | 15.34 | 10.33 | - | 10.55 | - | - | 73.46 |
| | A4 | Guangxi | 27.85 | - | - | 16.74 | - | - | 33.09 | 20.12 | - | 8.68 | - | - | 106.47 |
| | A5 | Yunnan | 24.62 | - | 3.14 | 17.21 | - | - | 31.85 | 23.57 | - | 11.54 | - | - | 111.92 |
| | A6 | Hubei | 54.89 | - | - | 12.15 | - | - | 20.56 | 13.06 | - | 9.38 | - | - | 110.05 |
| | A7 | Guangxi | 37.22 | - | 6.61 | 13.15 | - | - | 19.33 | 12.84 | - | - | - | - | 89.15 |
| | A8 | Guangdong | 21.54 | - | 3.80 | 20.33 | - | 9.68 | 24.85 | 18.72 | - | 11.73 | - | - | 110.66 |
| | A9 | Sichuan | 27.20 | - | 4.15 | 16.37 | 98.01 | 4.01 | 26.60 | 13.85 | - | 15.22 | - | - | 205.42 |
| | A10 | Hebei | 25.59 | 4.45 | 4.77 | 30.94 | 208.62 | 13.99 | 74.82 | 37.85 | - | 14.57 | - | - | 415.60 |
| | A11 | Jiangsu | 18.08 | - | 4.23 | 23.51 | 106.18 | 4.51 | 37.68 | 24.55 | - | 22.47 | - | 17.98 | 259.19 |
| | A12 | Hebei | 17.68 | 2.84 | 6.32 | 30.17 | 196.95 | 7.92 | 86.21 | 46.00 | - | 17.34 | - | - | 411.44 |
| | A13 | Sichuan | 19.23 | 3.09 | 8.25 | 18.18 | 61.22 | 3.00 | 15.64 | 10.77 | - | 8.84 | - | - | 148.22 |
| | A14 | Hebei | 27.62 | - | 5.68 | 11.90 | - | - | 27.71 | 20.63 | - | - | - | - | 93.55 |
| | A15 | Jiangsu | 22.03 | - | 3.26 | 13.80 | 116.76 | 4.11 | 38.10 | 18.87 | - | 10.72 | - | - | 227.66 |
| | A16 | Hunan | 25.74 | - | 4.20 | 15.41 | 86.13 | 7.63 | 13.28 | 8.32 | - | 8.38 | 17.87 | - | 186.96 |
| | A17 | Guangdong | 31.60 | - | 7.13 | 15.03 | - | 13.12 | 28.74 | 20.25 | - | 13.49 | 18.85 | - | 148.21 |
| | A18 | Shanghai | 17.98 | - | 3.00 | 11.04 | 65.03 | 6.31 | 11.83 | 8.43 | - | 8.17 | 18.88 | - | 150.67 |
| | A19 | Jiangxi | 21.30 | - | 5.20 | 16.85 | 130.53 | 5.70 | 23.29 | 11.89 | - | 11.00 | 19.99 | - | 245.76 |
| | A20 | Shandong | 24.37 | - | 6.84 | 16.12 | 66.41 | 4.65 | 20.06 | 11.84 | - | 9.88 | - | - | 160.18 |

| | | | | | | | | | | | | | | | |
|---|---|---|---|---|---|---|---|---|---|---|---|---|---|---|---|
| | A21 | Hunan | 29.02 | 3.49 | 7.78 | 27.62 | 136.35 | 8.77 | 36.92 | 39.02 | 9.60 | 27.83 | 25.29 | - | 351.69 |
| Perilla stems | B1 | Jiangsu | 9.05 | - | - | 2.52 | 7.09 | - | 2.51 | - | - | - | - | - | 21.18 |
| | B2 | Jiangsu | 5.51 | - | - | 2.55 | 10.63 | - | 2.68 | - | - | - | - | - | 21.37 |
| | B3 | Anhui | 13.85 | - | - | 2.54 | 7.97 | - | 2.75 | - | - | - | - | - | 27.11 |
| | B4 | Hebei | 6.85 | - | - | 2.80 | 10.81 | - | 4.96 | 4.57 | - | - | - | - | 30.00 |
| | B5 | Hubei | 17.20 | - | - | 3.78 | 11.40 | - | 5.45 | 3.04 | - | - | - | - | 40.88 |
| | B6 | Anhui | 10.93 | - | - | 2.14 | 5.12 | - | 2.13 | - | - | - | - | - | 20.32 |
| | B7 | Hubei | 7.77 | - | - | 2.14 | 4.78 | - | 2.58 | - | - | - | - | - | 17.28 |
| | B8 | Yunnan | 4.84 | - | - | - | 3.84 | - | - | - | - | - | - | - | 8.68 |
| | B9 | Guangdong | 4.11 | - | - | - | 2.92 | - | - | - | - | - | - | - | 7.02 |
| | B10 | Henan | 13.39 | - | - | 2.50 | 6.14 | - | 3.91 | 2.50 | - | - | - | - | 28.43 |
| | B11 | Anhui | 15.41 | - | - | 3.66 | 9.85 | - | 4.17 | 2.55 | - | - | - | - | 35.65 |
| | B12 | Hebei | 12.48 | - | - | 5.78 | 16.93 | 2.04 | 8.76 | 5.53 | - | - | - | - | 51.52 |
| | B13 | Hebei | 18.70 | - | - | 4.59 | 11.23 | - | 3.85 | - | - | - | - | - | 38.38 |
| | B14 | Guangxi | 12.02 | - | - | 2.34 | 6.69 | - | 3.82 | 3.18 | - | - | - | - | 28.06 |
| | B15 | Gansu | 16.59 | - | - | 3.42 | 11.59 | - | 5.78 | 3.88 | - | - | - | - | 41.26 |
| | B16 | Hunan | 10.01 | - | - | 2.66 | 12.28 | - | 3.73 | - | - | - | - | - | 28.69 |
| | B17 | Sichuan | 8.54 | - | - | 3.07 | 10.30 | - | 5.48 | 4.09 | - | - | - | - | 31.47 |
| | B18 | Guangdong | 9.92 | - | - | 2.72 | 17.80 | 2.04 | 3.88 | 2.08 | - | - | - | - | 38.43 |
| | B19 | Guangdong | 8.99 | - | - | 2.95 | 23.54 | - | 4.35 | 2.43 | - | - | - | - | 42.26 |
| | B20 | Hunan | 10.61 | - | - | 3.01 | 15.49 | - | 4.58 | 3.25 | - | - | - | - | 36.94 |
| Perilla seeds | C1 | Sichuan | 18.18 | - | 2.82 | - | 2.86 | - | - | - | - | - | - | - | 23.87 |
| | C2 | Anhui | 31.00 | - | 2.20 | 3.22 | 25.68 | - | 10.39 | 6.87 | - | - | - | - | 79.36 |
| | C3 | Shanxi | 20.80 | - | 2.31 | - | 6.71 | - | - | - | - | - | - | - | 29.82 |
| | C4 | Heilongjiang | 31.12 | - | 2.53 | - | 5.33 | - | - | - | - | - | - | - | 38.98 |
| | C5 | Guangxi | 144.82 | - | 3.14 | 3.13 | 21.14 | - | 4.22 | 3.54 | - | - | - | - | 180.00 |
| | C6 | Heilongjiang | 22.47 | - | 3.58 | 2.91 | 17.10 | - | 5.18 | 4.93 | - | - | - | - | 56.18 |
| | C7 | Hubei | 42.73 | - | 3.23 | 2.08 | 13.21 | - | 3.56 | 2.80 | - | - | - | - | 67.61 |
| | C8 | Yunnan | 43.55 | - | 4.16 | 3.66 | 19.45 | - | 6.83 | 5.24 | - | - | - | - | 82.88 |
| | C9 | Hubei | 28.44 | - | 3.85 | 3.40 | 12.58 | - | 2.22 | - | - | - | - | - | 50.49 |
| | C10 | Sichuan | 38.69 | - | 3.93 | 4.77 | 19.05 | - | 3.39 | 4.16 | - | - | - | - | 73.97 |
| | C11 | Hubei | 37.15 | - | 16.06 | 17.59 | 48.31 | - | 3.62 | 2.11 | - | - | - | - | 124.84 |
| | C12 | Jiangsu | 22.61 | - | 3.53 | 3.49 | 12.95 | - | - | - | - | - | - | - | 42.58 |
| | C13 | Shanxi | 18.46 | - | 10.30 | 9.71 | 21.94 | - | - | - | - | - | - | - | 60.41 |
| | C14 | Hebei | 9.41 | - | 2.83 | - | 3.00 | - | - | - | - | - | - | - | 15.24 |
| | C15 | Shandong | 50.68 | 2.46 | 2.70 | 5.67 | 33.80 | 2.83 | 10.26 | 6.88 | - | - | - | - | 115.27 |
| | C16 | Anhui | 34.34 | 2.59 | 5.58 | 7.09 | 29.70 | 2.42 | 7.90 | 5.11 | - | - | - | - | 94.72 |
| | C17 | Jiangsu | 23.59 | 2.24 | 3.31 | 3.24 | 12.88 | - | 3.13 | 2.22 | - | - | - | - | 50.62 |
| | C18 | Shanxi | 21.79 | - | 6.29 | 4.51 | 15.04 | - | 2.57 | - | - | - | - | - | 50.19 |
| | C19 | Shaanxi | 24.51 | - | 2.26 | 2.61 | 7.93 | - | - | - | - | - | - | - | 37.31 |
| | C20 | Shaanxi | 32.44 | - | 3.31 | 3.25 | 9.68 | - | - | - | - | - | - | - | 48.68 |
| | C21 | Guangdong | 46.36 | - | 5.82 | 5.74 | 31.22 | 2.16 | 7.25 | 4.74 | - | - | - | - | 103.29 |
| | C22 | Guangdong | 17.66 | - | 2.68 | 2.44 | 5.86 | - | - | - | - | - | - | - | 28.66 |
